# Prone position reduces the risk of patients with mild or moderate COVID-19 progressing to severe or even critical cases: a retrospective study

**DOI:** 10.1186/s40001-022-00776-y

**Published:** 2022-08-12

**Authors:** Chuan-cai Xu, Jia-li Xu, Xiao-fei Wang, Shen Meng, Sheng Ye, Xiao-miao Tang, Wei Lei

**Affiliations:** Department of Pulmonary and Critical Care Medicine, The First Affiliated Hospital of Soochow University, 899#, Pinghai Road, Gusu District, Suzhou, 215006 Jiangsu Province China

**Keywords:** COVID-19, Prone position, Prone position ventilation, Progression, Mild, Moderate

## Abstract

**Background:**

To investigate whether prone position can reduce the risk of patients with mild or moderate COVID-19 who progress to severe or critical illness.

**Methods:**

The prone position group was treated in prone position on the day of admission in addition to conventional treatment. Indicators such as saturation of pulse oximetry (SpO_2_), heart rate, blood pressure, respiratory rate, and prone position-related adverse events were recorded before prone ventilation, 5 min after prone position and 30 min after prone position. Meanwhile, the cases of severe and critical patients, the percentage of transformation and the final clinical outcome of this group were analyzed. Conversion rates and mortality were calculated for patients with mild or moderate COVID-19 retrieved from the database who received only conventional care without combined prone positioning as control group.

**Results:**

(1) A total of 34 patients were included in prone position group. There were significant differences in SpO_2_ between the first 4 days after admission and the day of discharge (*F* = 3.17, *P* < 0.001). (2) The main complications were back and neck muscle soreness (55.9%), followed by abdominal distension (8.9%). (3) In control group, a total of 4873 cases of mild and moderate patients were included from 19 literatures, with an average deterioration rate of 22.7% and mortality rate of 1.7%. (4) In prone position group, there were no severe or critical transformation cases and also no death cases. The prone position group had a significantly lower deterioration rate when compared with the control group (χ^2^ = 9.962, *P* < 0.01).

**Conclusion:**

Prone position improves SpO_2_ in patients with mild or moderate COVID-19. It can also reduce the percentage of mild or moderate patients progressing to severe or critical patients. The application of prone position is a simple, feasible, safe and effective treatment method in such patients.

## Background

COVID-19 can cause a subset of patients to develop acute respiratory distress syndrome (ARDS) [1, 2]. Prone position ventilation is a mechanical ventilation method that uses various means to put patients in prone position, and its therapeutic status in ARDS has been widely recognized [3, 4]. As a result of COVID-19 outbreak, there are an increasing number of studies on the use of prone position ventilation in severe and critically ill patients, showing that prone position ventilation can also improve oxygenation in COVID-19 patients. However, more research is still needed to confirm whether it can reduce mortality and improve patient outcomes [5–8]. Recent studies have shown that prone position combined with oxygen inhalation by mask or high-flow nasal cannula oxygen therapy (HFNC) or non-invasive positive pressure ventilation has the clinical efficacy in reducing the rate of endotracheal intubation and mortality in the treatment of severe COVID-19 patients. Meanwhile, studies have shown that early prone position has better clinical efficacy than late prone position [9, 10].

COVID-19 patients can be classified into mild, moderate, severe and critical according to the severity of their illness [11, 12]. Studies have found that about 0 to 45.0% of mild or moderate patients can progress to severe or critical patients [13–31]. Once progressed to severe or critical patients, the place of treatment needs to be transferred from ordinary wards or Fangcang shelter hospitals to intensive care unit (ICU) wards, which will greatly increase the difficulty of treatment and the medical resources needed. Even worse the mortality rate of critically ill patients is as high as 49% [32–34].

There are few reports on whether the combination of prone position therapy with conventional therapy can reduce the percentage of patients with mild or moderate COVID-19 who develop to severe or critical illness, and thus reduce patient mortality, especially with regard to the Delta subtype of COVID-19. Therefore, we recorded the changes of vital signs such as saturation of pulse oximetry (SpO_2_) and the occurrence of complications in patients with mild or moderate COVID-19 after prone position, and the percentage of patients progressing to severe or critical COVID-19. We analyzed and compared the obtained results with the control group (reported cases without prone position and only received conventional treatment), and the clinical value of prone position in mild-to-moderate patients was preliminarily explored.

## Material and methods

### Diagnostic criteria

According to the National Health Commission's Novel Coronavirus Protocol for the diagnosis and treatment of pneumonia (Trial Version 8) [12], the diagnostic criteria includes following aspects: epidemiological history, clinical manifestations are fever and/ or respiratory symptoms, chest imaging shows pneumonia, real-time fluorescence RT-PCR is used to detect positive SARS-CoV-2 Delta nucleic acid in nasal swabs or pharyngeal swabs.

### General information

**Fig. 1:**
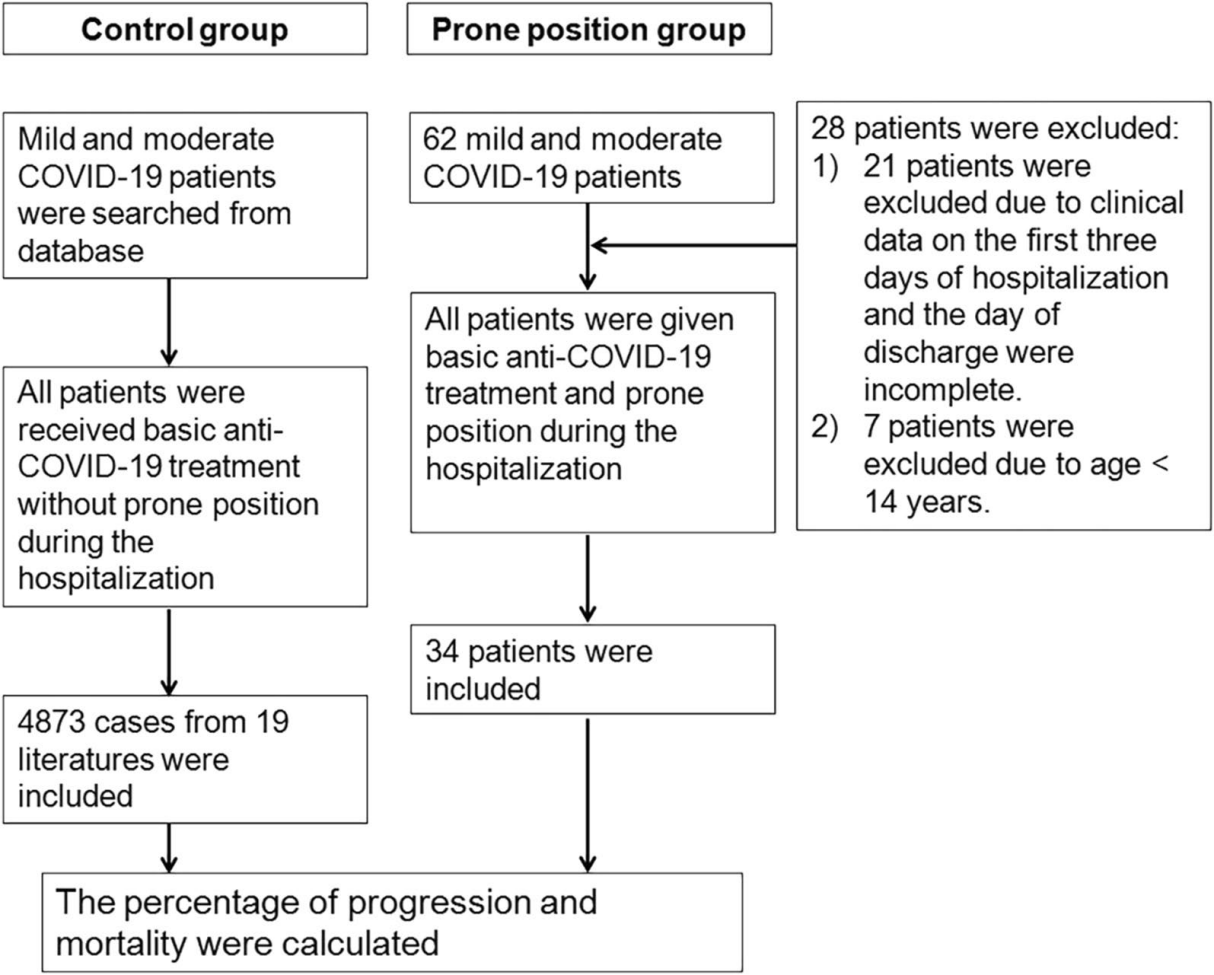
Flowchart of patients enrollment

Prone position group: clinical data of 62 patients with mild and moderate COVID-19 admitted to Yangzhou Third People's Hospital from July to August 2021 were collected. All patients were given traditional Chinese medicine anti-viral therapy and symptomatic supportive treatment upon admission, and all patients were in prone position on the day of admission. Among these patients, 21 patients were transferred to other hospitals for treatment (hospital duration ≤ 3 days, no follow-up clinical data), and 7 patients (< 14 years) did not undergo chest computed tomography (CT) examination or were not reviewed. The above 28 patients were excluded due to lack of clinical data. Thus, a total of 34 patients with complete clinical data were finally enrolled in the study, the inclusion of patients in prone position group are shown in Fig. 1. They were 14 to 83 years old and spent 8 to 12 h a day in prone position. Chest CT examination and vital signs (SpO_2_, heart rate, blood pressure and respiratory rate) were performed at admission, and chest CT was reviewed later.

Control group: The related data were searched from database including Cochrane Library, PubMed, Excerpta Medica Database (EMBASE), China National Knowledge Infrastructure (CNKI) and Wanfang Databases. Search keywords are COVID-19, mild, moderate and progression. Search languages are English and Chinese (with English abstract required). The search deadline was December 31, 2021. During hospitalization, only conventional treatment was performed in these patients without prone position, and duplicate references were excluded. The number of patients with mild and moderate COVID-19 at the beginning of treatment, and the number of patients with severe and critical COVID-19 at the later stage were counted, and the percentage of progression and mortality were calculated. The inclusion of patients in control group are shown in Fig. 1.

### Methods of prone position

All patients had independent activity ability. The operation method is as follows: patients face down, arms on both sides of the body, legs straight, a soft pillow can be put under the chest, hip and ankle, head biased to one side, their own initiative to choose a most comfortable decubitus position.

### Observation indicators

SpO_2_, heart rate, blood pressure, respiratory rate, prone position-related adverse events, changes in clinical classification of COVID-19 and the final clinical outcome (mortality) were recorded before the first prone position, 5 min after prone position, 30 min after prone position in the first 4 days after admission and the day of discharge.

### Statistical methods

SPSS 26.0 was used for data analysis. The measurement data were expressed as mean ± standard deviation, and the counting data were expressed as the number of cases and rate. Analysis of variance (ANOVA) was used for data comparison among multiple groups. Chi-square test was used for the data rates between two groups. *P* < 0.05 means the difference is statistically significant.

## Results

### Influence of prone position on SpO_2_

**Fig. 2:**
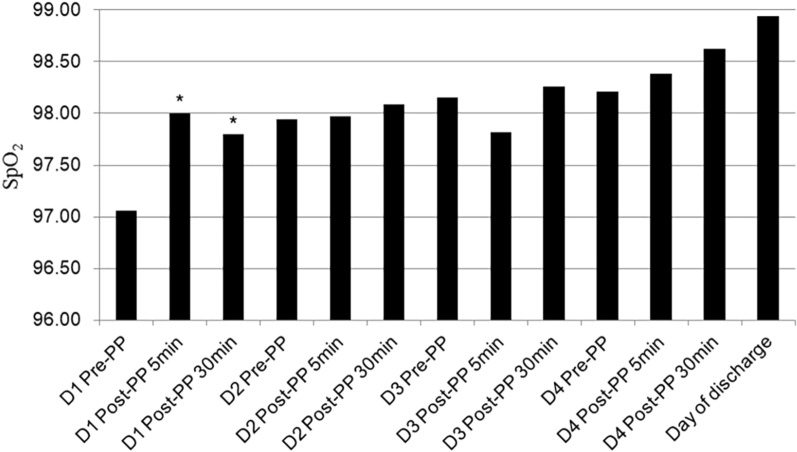
Influence of prone position on SpO_2_. SpO_2_, saturation of pulse oximetry; Pre-PP, before prone position; Post-PP, after prone position; D1, D2, D3, D4, the first 4 days after admission. There was significant difference in SpO_2_ between the first 4 days after admission and the day of discharge. *, compared with D1 Pre-PP, *P* < 0.05, On the first day of admission, the level of SpO_2_ in the prone position was higher after 5 min and 30 min than before prone position.

There was a significant difference in SpO_2_ between the first 4 days after admission and the day of discharge (*F* = 3.17, *P* < 0.001). SpO_2_ was the lowest on admission and the highest on discharge day. On the first day of admission, the level of SpO_2_ in the prone position was higher after 5 min and 30 min than before, while there was no significant difference between 5 and 30 min after the prone position. On the 2nd, 3rd and 4th days after admission, there was no significant difference in SpO_2_ before and after prone position (all *P* > 0.05) (Fig. 2).

### Influence of prone position on respiratory rate, heart rate and blood pressure (systolic and diastolic)

**Fig. 3:**
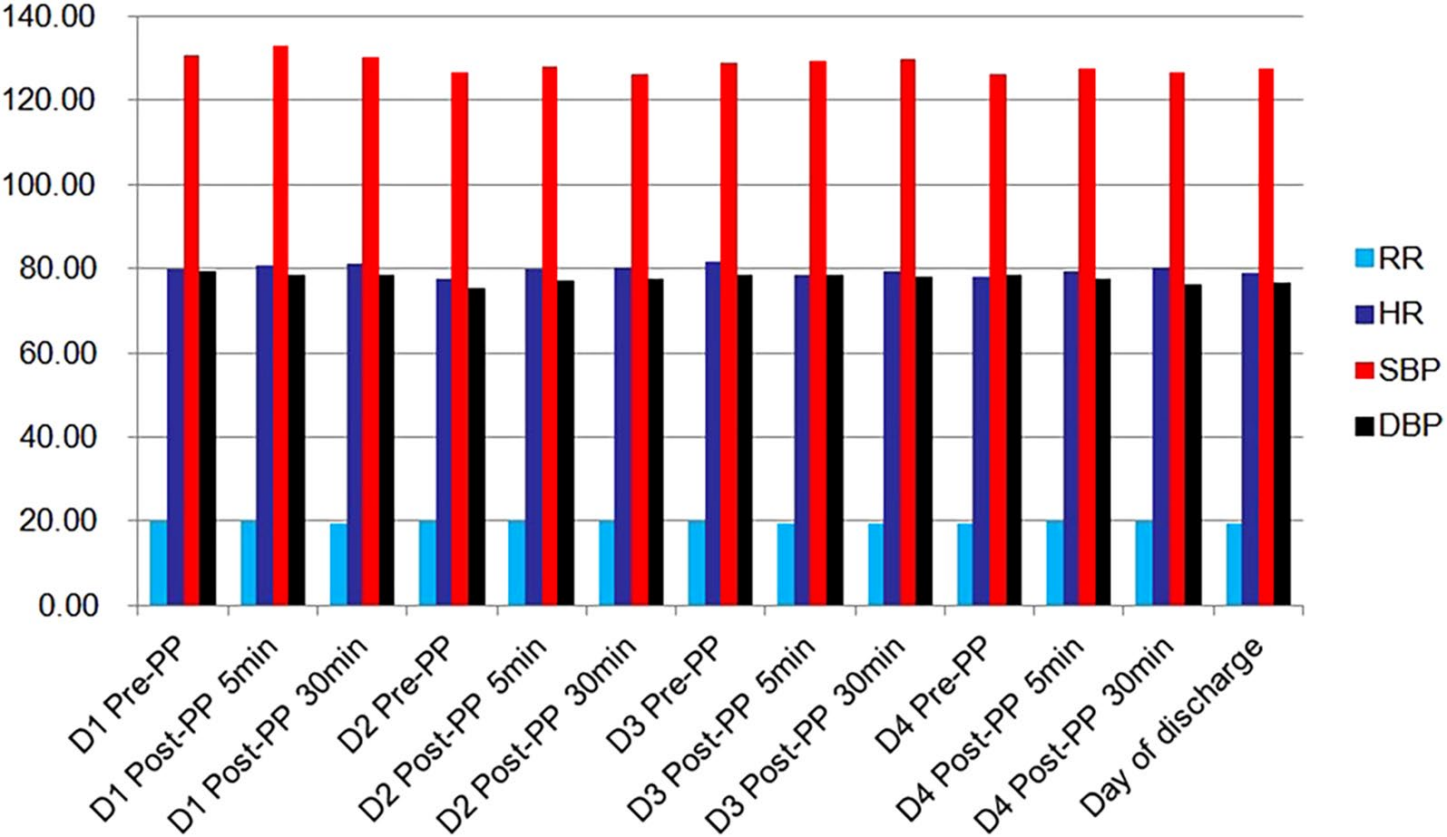
Influence of prone position on respiratory rate, heart rate and blood pressure. Pre-PP, before prone position; Post-PP, after prone position; D1, D2, D3, D4, the first 4 days after admission; RR, respiratory rate; HR, heart rate; SBP, systolic blood pressure; DBP, diastolic blood pressure. There were no statistically significant differences in RR, HR, SBP and DBP between patients in the first 4 days after admission and on the day of discharge (*F* = 0.641, 0.573, 0.671 and 0.386, respectively, *P* > 0.05).

There were no statistically significant differences in respiratory rate, heart rate, systolic blood pressure and diastolic blood pressure between patients in the first 4 days after admission and on the day of discharge (*F* = 0.641, 0.573, 0.671 and 0.386, respectively, all *P* > 0.05) (Fig. 3).

### Prone position-related adverse events

The main complication was back and neck muscle soreness, accounting for 55.9% (19/34), which was considerably relieved or disappeared in most patients within 3 days. Abdominal distension occurred in 8.9% (3/34) of patients and was relieved by supine position or exercise. There was no indwelling of deep vein, catheter, endotracheal intubation, etc., so there were no complications such as catheter displacement. Pressure ulcers, facial edema, optic nerve damage, pneumothorax and other complications were also not observed.

### Progression and mortality in prone position group

There was a total of 34 patients in prone position group, among which 3 patients progressed from mild-to-moderate illness. The SARS-CoV-2 Delta nucleic acid test of all patients ultimately turned negative and patients were then admitted to the rehabilitation hospital for follow-up treatment, and no death cases occurred. No patient progressed to severe or critical cases, with a rate of deterioration of 0%. The mortality rate in this group was also 0%.

### Progression of patients in control group

The literature was searched, and duplicates were removed. In total, 19 Chinese and English literatures that met the requirements were identified. Among these 19 literatures, there were 4873 cases of mild or moderate patients, of which 1106 cases progressed to severe or critical cases. The deterioration rate from low to high were 0%, 5.7%, 6.0%, 8.3%, 10.9%, 13.7%, 16.1%, 16.4%, 19.0%, 20.3%, 21.7%, 23.2%, 27.0%, 29.4%, 31.6%, 34.9%, 36.9%, 40.9% and 45.0%. The average deterioration rate was 22.7%. There were 69 deaths and the mortality rate was 1.7% (Table 1).

**Table 1:** The progression and mortality of mild or moderate patients in control group

Literature	Country	Time of case occurrence	Number of mild or moderate	Number of progression	Progression rate (%)	Number of deaths	Mortality (%)
Wang et al. [13]	Tianjin, China	January to March 2020	95	22	23.2	0	0
Higuchi et al. [14]	Japan	February to June 2020	37	0	0	0	0
Iijima et al. [15]	Japan	April to May 2020	55	6	10.9	NR	NR
Cheng et al. [16]	Hubei, China	January to March 2020	456	205	45.0	46	10.1
Wang et al. [17]	Hubei, China	February to April 2020	1758	474	27.0	9	0.5
Long et al. [18]	Hubei, China	January to February 2020	253	48	19.0	5	2.0
Jiang et al. [19]	Hubei, China	January to April 2020	213	35	16.4	NR	NR
Zhang et al. [20]	Guangdong, China	January to February 2020	12	1	8.3	0	0
Li et al. [21]	Anhui, China	January to February 2020	65	24	36.9	0	0
Liang et al. [22]	Guangdong, China	January to February 2020	204	28	13.7	0	0
Zhang et al. [23]	Sichuan, China	2020	98	31	31.6	0	0
Lang et al. [24]	Sichuan, China	January to February 2020	17	5	29.4	0	0
Liu et al. [25]	Hubei, China	January to February 2020	62	10	16.1	6	9.7
Duan et al. [26]	Chongqing, China	January to February 2020	348	20	5.7	NR	NR
Lv et al. [27]	Beijing, China	January to February 2020	64	13	20.3	0	0
Luo et al. [28]	Jiangsu, China	January to February 2020	597	36	6.0	0	0
Zhao et al. [29]	Hubei, China	February 2020	172	60	34.9	NR	NR
Wang et al. [30]	Shenzhen, China	January to February 2020	323	70	21.7	3	0.9
Lu et al. [31]	Shanghai, China	January to February 2020	44	18	40.9	0	0

*NR*, not recorded

### Effect of prone position on conversion from mild or moderate to severe or critical patients

The deterioration rate was 0% in prone group and 22.7% in control group. The proportion of patients with mild or moderate illness progression to severe or critical disease in prone group was significantly lower than that in control group, and the difference was statistically significant (χ^2^ = 9.962, *P* < 0.01) (Table 2).

**Table 2:** Effect of prone position on progression from mild or moderate cases to severe or critical cases

	Control group	Prone position group	χ^2^ value	*P* value
Mild or moderate cases	3767	34	9.962	0.002
Severe or critical cases	1106	0		
Total	4873	34		
Progression rate	22.7%	0%		

### Effect of prone position on mortality between prone position group and control group

The mortality rate was 0% in prone position group and 1.7% in control group. There was no statistically significant difference in mortality between the two groups. (χ^2^ = 1.154, *P* > 0.05) (Table 3).

**Table 3:** Effect of prone position on mortality between prone position group and control group

	Control group	Prone position group	χ^2^ value	*P* value
Cases	4085	34	1.154	0.283
Deaths	69	0		
Mortality	1.7%	0%		

## Discussion

According to the severity of the disease, COVID-19 can be divided into four levels: (1) mild—the clinical symptoms are mild and there is no pneumonia on imaging; (2) moderate—fever, respiratory symptoms, imaging manifestations of pneumonia; (3) severe—adults meet any of the following criteria: a. shortness of breath; respiratory rates ≥ 30 times /min; b. SpO_2_ ≤ 93% when inhaling air in resting state; c. arterial partial pressure of oxygen/ oxygen concentration ≤ 300 mmHg; d. clinical symptoms are progressively worse, and lung imaging shows obvious progression (more than 50%) within 24–48 h; (4) critical—meeting one of the following criteria: a. respiratory failure, and the need for mechanical ventilation treatment; b. slip into shock; c. ICU treatment is required for other organ failures [12]. In the course of clinical treatment, it has been found that some patients with mild and moderate COVID-19 can evolve into severe or even critical COVID-19 in a short period of time [13–31]. The principles of treatment for patients with different severity vary greatly. In addition to anti-viral therapy, bed rest and strong nutritional support are the main treatment options for mild and moderate patients. In addition, such patients also need to pay attention to water and electrolyte balance, maintain internal environment stability, and closely monitor vital signs such as SpO_2_. Effective oxygen therapy measures, including nasal cannula, mask oxygen inhalation, and HFNC, were administered when necessary. For the treatment of severe and critically ill patients, in addition to the above treatment measures, mechanical ventilation and other organ function support means are also needed. It can be seen that severe and critical patients are much more difficult to treat than mild and moderate patients [12].

Studies have shown that prone position ventilation can promote the homogeneity of gas distribution, improve the ratio of ventilatory blood flow and oxygenation function in ARDS patients. Besides, prone position ventilation can also promote sputum drainage, reduce the incidence of ventilator-associated pneumonia, improve heart function, reduce mediastinal and cardiac compression of the lungs and other physiological effects, thereby reducing mortality [3, 35, 36]. Prone position ventilation can improve oxygenation in critical COVID-19 patients, but whether it can reduce mortality is still controversial [5, 6, 37]. Prone position is a position in which the patient is treated by remaining prone without endotracheal intubation. Some studies have found that for severe patients, prone position combined with mask oxygen or HFNC or non-invasive positive pressure ventilation can reduce the probability of endotracheal intubation and ultimately reduce patient mortality [38–42]. Other studies have shown that prone position may be more effective when performed early than later [10, 43]. It has also been shown that the earlier patients are admitted to hospital for intervention, the less likely mild and moderate cases will progress to severe and critical [44]. However, there are few studies concerning whether it is possible to reduce the percentage of conversion to severe and critical cases if prone position was performed on the day of admission in mild and moderate patients.

In our study, all mild and moderate patients were treated in prone position (prone position group) on the basis of conventional treatment. We calculated the percentage of conversion in this group, 3 of 34 patients progressed from mild to moderate, but no patients progressed to serve or critical cases, in other words, the conversion rate in this group was 0%, and the mortality was 0% either. Unfortunately, due to the study was non-interventional, we were unable to randomly assign a group of patients with mild or moderate disease to conventional treatment. Therefore, the deterioration rate of control group was obtained through literature review. The results of literature survey showed that the deterioration rate of control group to severe or critical cases was 0% to 45.0%, the average deterioration rate was 22.7%, and the mortality rate was 1.7% [13–31]. The results showed that the percentage of patients progressing to severe or critical cases was significantly lower in prone position group than in control group. The results suggested that the prone position may be helpful in reducing the percentage of patients with mild and moderate disease progressing to severe and critical disease. Our study found that the prone position did not reduce the mortality in patients with mild or moderate patients, and we speculate that this may be related to the small sample size of prone position group, which needs to be confirmed by subsequent studies with larger sample sizes. The results of our study showed that SpO_2_ improved gradually after treatment in prone position, especially after the first day of admission. There were no changes in respiratory rate, heart rate, blood pressure (systolic and diastolic) before and after prone position. This is basically consistent with the research results of other scholars [41, 43].

In addition, during the entire hospital stay, 55.9% of patients presented with back and neck muscle soreness after prone position, which basically relieved or disappeared within 3 days. Abdominal distention occurred in 8.9% of patients and was relieved by supine position or exercise. There were no indwelling of deep vein, catheter, endotracheal intubation, etc., so there were no complications such as catheter displacement [45]. Pressure ulcers, facial edema, optic nerve damage, pneumothorax and other complications were also not observed. Majority of patients only required the guidance of medical staff for the first time of prone position, the rest of the time were completed by themselves, further revealing the prone position clinical implementation is simple and easy. In some critically ill patients, prone positioning was often supplemented with sedation [46], however in our study, no patients required sedation. In conclusion, prone position can improve patients’ oxygenation and reduce the progressing rate during the clinical treatment of mild and moderate patients. In addition, prone position in such patients is relatively safe, with almost no serious complications and little effort from medical staff, which is worthy of further promotion.

Patients with COVID-19 are prone to abnormal coagulation, especially in severe and critical patients who spend a long time in bed and are prone to venous thrombosis and even pulmonary embolism [47–49]. There were no cases of venous thrombosis or pulmonary embolism in our study. But we also have some questions that need to be answered by subsequent studies: (1) Do all mild and moderate patients need prone position? (2) How many hours per day is prone position required without increasing the risk of blood clots? (3) How long should prone position treatment last?

## Limitations

(1) This study was a single-center observational study with a small sample size, which needs to be further confirmed by a multi-center large sample size study. (2) The study did not set a control group at the same time. Data in the literature were captured and compared, so the baseline status of patients in control group may be inconsistent with that of the study population, resulting in statistical bias. (3) No blood gas analysis was performed, and therefore no improvement in oxygenation was compared from the perspective of arterial partial pressure of oxygen. (4) Due to the epidemic situation, only the prone position for 5 min and 30 min were set, and no longer time points such as 120 min were set for observation. (5) All viruses in prone group were infected with the Delta subtype, while those in control group were mainly infected with α, β or γ subtypes. Different virus subtypes might affect the study results.

## Conclusions

For patients with mild and moderate COVID-19, the combination of the prone position with the conventional treatment can improve oxygenation, but it has little impact on the patients’ heart rate, blood pressure and respiratory rate. Also, no significant complications occurred. It can reduce the percentage of mild or moderate patients progressing to severe or critical patients. The use of prone position is a simple, feasible, safe and efficient treatment method in such patients. The conclusions need to be further confirmed by a multi-center larger sample size study.

## Data Availability

The datasets used and/or analyzed during the current study are available from the corresponding author on reasonable request.

## Abbreviations

SpO_2_: Saturation of pulse oximetry
ARDS: Acute respiratory distress syndrome
HFNC: High-flow nasal cannula oxygen therapy
CT: Computed tomography
ICU: Intensive care unit

## References

[1] Montenegro F, Unigarro L, Paredes G, Moya T, Romero A, Torres L (2021). Acute respiratory distress syndrome (ARDS) caused by the novel coronavirus disease (COVID-19): a practical comprehensive literature review. Expert Rev Respir Med.

[2] El-Solh AA, Meduri UG, Lawson Y, Carter M, Mergenhagen KA (2021). Clinical course and outcome of COVID-19 acute respiratory distress syndrome: data from a national repository. J Intensive Care Med.

[3] Guérin C, Reignier J, Richard JC, Beuret P, Gacouin A, Boulain T (2013). Prone positioning in severe acute respiratory distress syndrome. N Engl J Med.

[4] Munshi L, Del Sorbo L, Adhikari NKJ, Hodgson CL, Wunsch H, Meade MO (2017). Prone position for acute respiratory distress syndrome a systematic review and meta-analysis. Ann Am Thorac Soc.

[5] Vollenberg R, Matern P, Nowacki T, Fuhrmann V, Padberg JS, Ochs K (2021). Prone position in mechanically ventilated COVID-19 patients: a multicenter study. J Clin Med.

[6] Langer T, Brioni M, Guzzardella A, Carlesso E, Cabrini L, Castelli G (2021). Prone position in intubated, mechanically ventilated patients with COVID-19: a multi-centric study of more than 1000 patients. Crit Care.

[7] Stilma W, van Meenen DMP, Valk CMA, de Bruin H, Paulus F, Neto AS (2021). Incidence and practice of early prone positioning in invasively ventilated COVID-19 patients-insights from the PRoVENT-COVID observational study. J Clin Med.

[8] Gleissman H, Forsgren A, Andersson E, Lindqvist E, Lipka Falck A, Cronhjort M (2021). Prone positioning in mechanically ventilated patients with severe acute respiratory distress syndrome and coronavirus disease 2019. Acta Anaesthesiol Scand.

[9] Ehrmann S, Li J, Ibarra-Estrada M, Perez Y, Pavlov I, McNicholas B (2021). Awake prone positioning for COVID-19 acute hypoxaemic respiratory failure: a randomised, controlled, multinational, open-label meta-trial. Lancet Respir Med.

[10] Kaur R, Vines DL, Mirza S, Elshafei A, Jackson JA, Harnois LJ (2021). Early versus late awake prone positioning in non-intubated patients with COVID-19. Crit Care.

[11] Report of the WHO-China joint mission on coronavirus disease 2019 (COVID-19). 2020; https://www.who.int/docs/defaultsource/coronaviruse/who-china-joint-mission-on-covid-19-final-report.pdf. Accessed 16 Jun 2020.

[12] National Health Commission of the People's Republic of China. COVID-19 diagnosis treatment protocol (8th trial version). http://www.gov.cn/zhengce/zhengceku/2021-04/15/content_5599795.htm (in Chinese)

[13] Wang HR, Yu HZ, Li L, Hua JN, Wang X, Zhou YY (2021). Risk factors of progression from mild to severe COVID-19 and the predictive value of age-adjusted Charlson complication index. Shandong Med J.

[14] Higuchi T, Nishida T, Iwahashi H, Morimura O, Otani Y, Okauchi Y (2021). Early clinical factors predicting the development of critical disease in Japanese patients with COVID-19: a single-center, retrospective, observational study. J Med Virol.

[15] Iijima Y, Okamoto T, Shirai T, Mitsumura T, Sakakibara R, Honda T (2021). MuLBSTA score is a useful tool for predicting COVID-19 disease behavior. J Infect Chemother.

[16] Cheng B, Hu J, Zuo X, Chen J, Li X, Chen Y (2020). Predictors of progression from moderate to severe coronavirus disease 2019: a retrospective cohort. Clin Microbiol Infect.

[17] Wang W, Shen M, Tao Y, Fairley CK, Zhong Q, Li Z (2021). Elevated glucose level leads to rapid COVID-19 progression and high fatality. BMC Pulm Med.

[18] Long L, Zeng X, Zhang X, Xiao W, Guo E, Zhan W (2020). Short-term outcomes of COVID-19 and risk factors for progression. Eur Respir J.

[19] Jiang H, Li J, Luo JH (2020). Prognostic value of different inflammation-based score indicators on COVID-19 from normal type to severe type. Mil Med J S Chin.

[20] Zhang WD, Lu SR, Zhang MF, Zheng HB, Huang YH, Chen SZ (2020). Correlation between hyponatremia and the severity of coronavirus disease 2019. Chin Crit Care Med.

[21] Li DM, Liu CM, Liu JH, Hu JF, Yang YL, Zhou YF (2020). Analysis of the risk factors of the common type evolving into severe type COVID-19. J Bengbu Med Coll.

[22] Liang ZW, Luo A, Wen CY, Feng LZ, Xiang FF, Li PH (2020). Risk factors to predict the occurrence of critical illness in patients with COVID-19. Inter J Epidemiol Infect Dis.

[23] Zhang LH, Chen H, Zhang L, Chen XR (2021). Risk factors for progression to severe disease in patients with mild COVID-19. Mod Prev Med..

[24] Lang MJ, Zhang Z, Fu GQ, Huang X, Liu X, Li J (2020). Clinical features and laboratory indicators in progression of corona virus disease 2019 to severe type. Chin J TCM WM Crit Care.

[25] Liu J, Liu Z, Jiang W, Wang J, Zhu M, Song J (2021). Clinical predictors of COVID-19 disease progression and death: analysis of 214 hospitalised patients from Wuhan China. Clin Respir J.

[26] Duan J, Wang X, Chi J, Chen H, Bai L, Hu Q (2020). Correlation between the variables collected at admission and progression to severe cases during hospitalization among patients with COVID-19 in Chongqing. J Med Virol.

[27] Lv Z, Guan C, Yan S, Cui T, Zhou A, Xie R (2020). Value of CT findings in predicting transformation of clinical types of COVID-19. Chin J Radiol.

[28] Luo H, Liu S, Wang Y, Mortimer K, Ju S, Yang Y (2020). Disease progression in patients with COVID-19: a retrospective cohort study in China. Int J Tuberc Lung Dis.

[29] Zhao C, Bai Y, Wang C, Zhong Y, Lu N, Tian L (2021). Risk factors related to the severity of COVID-19 in Wuhan. Int J Med Sci.

[30] Wang F, Qu M, Zhou X, Zhao K, Lai C, Tang Q (2020). The timeline and risk factors of clinical progression of COVID-19 in Shenzhen, China. J Transl Med.

[31] Lu Y, Sun K, Guo S, Wang J, Li A, Rong X (2020). Early warning indicators of severe COVID-19: a single-center study of cases from Shanghai, China. Front Med (Lausanne).

[32] Wu Z, McGoogan JM (2020). Characteristics of and important lessons from the coronavirus disease 2019 (COVID-19) outbreak in China: summary of a report of 72314 cases from the Chinese center for disease control and prevention. JAMA.

[33] Yang X, Yu Y, Xu J, Shu H, Xia J, Liu H (2020). Clinical course and outcomes of critically ill patients with SARS-CoV-2 pneumonia in Wuhan, China: a single-centered, retrospective, observational study. Lancet Respir Med.

[34] Azarkar Ghodsiyeh, Osmani Freshteh (2021). Clinical characteristics and risk factors for mortality in COVID-19 inpatients in Birjand, Iran: a single-center retrospective study. European Journal of Medical Research.

[35] Kallet RH (2015). A comprehensive review of prone position in ARDS. Respir Care.

[36] Gattinoni L, Busana M, Giosa L, Macrì MM, Quintel M (2019). Prone positioning in acute respiratory distress syndrome. Semin Respir Crit Care Med.

[37] Scaramuzzo G, Gamberini L, Tonetti T, Zani G, Ottaviani I, Mazzoli CA (2021). Sustained oxygenation improvement after first prone positioning is associated with liberation from mechanical ventilation and mortality in critically ill COVID-19 patients: a cohort study. Ann Intensive Care.

[38] Rosén J, von Oelreich E, Fors D, Fagerlund MJ, Taxbro K, Skorup P (2021). Awake prone positioning in patients with hypoxemic respiratory failure due to COVID-19: the PROFLO multicenter randomized clinical trial. Crit Care.

[39] Pb S, Mittal S, Madan K, Mohan A, Tiwari P, Hadda V (2021). Awake prone positioning in non-intubated patients for the management of hypoxemia in COVID-19: a systematic review and meta-analysis. Monaldi Arch Chest Dis.

[40] Pavlov I, He H, McNicholas B, Perez Y, Tavernier E, Trump MW (2021). Awake prone positioning in non-intubated patients with acute hypoxemic respiratory failure due to COVID-19. Respir Care.

[41] Ferrando C, Mellado-Artigas R, Gea A, Arruti E, Aldecoa C, Adalia R (2020). Awake prone positioning does not reduce the risk of intubation in COVID-19 treated with high-flow nasal oxygen therapy: a multicenter, adjusted cohort study. Crit Care.

[42] Ehrmann S, Li J, Ibarra-Estrada M, Perez Y, Pavlov I, McNicholas B (2021). Awake prone positioning for COVID-19 acute hypoxaemic respiratory failure: a randomised, controlled, multinational, open-label meta-trial. Lancet Respir Med.

[43] Liu X, Liu H, Lan Q, Zheng X, Duan J, Zeng F (2021). Early prone positioning therapy for patients with mild COVID-19 disease. Med Clin (Barc).

[44] Peng L, Lv QQ, Yang F, Wu XM, Zhang CC, Wang YQ (2021). The interval between onset and admission predicts disease progression in COVID-19 patients. Ann Transl Med.

[45] Lee JM, Bae W, Lee YJ, Cho Y (2014). The efficacy and safety of prone positional ventilation in acute respiratory distress syndrome: updated study-level meta-analysis of 11 randomized controlled trials. Crit Care Med.

[46] Taboada M, Baluja A, Santos LD, González I, Veiras S, Caruezo V (2021). Effectiveness of dexmedetomidine combined with high flow nasal oxygen and long periods of awake prone positioning in moderate or severe COVID-19 pneumonia. J Clin Anesth.

[47] Teimury A, Khameneh MT, Khaledi EM (2022). Major coagulation disorders and parameters in COVID-19 patients. Eur J Med Res.

[48] Helms J, Tacquard C, Severac F, Leonard-Lorant I, Ohana M, Delabranche X (2020). High risk of thrombosis in patients with severe SARS-CoV-2 infection: a multicenter prospective cohort study. Intensive Care Med.

[49] Connors JM, Levy JH (2020). COVID-19 and its implications for thrombosis and anticoagulation. Blood.

